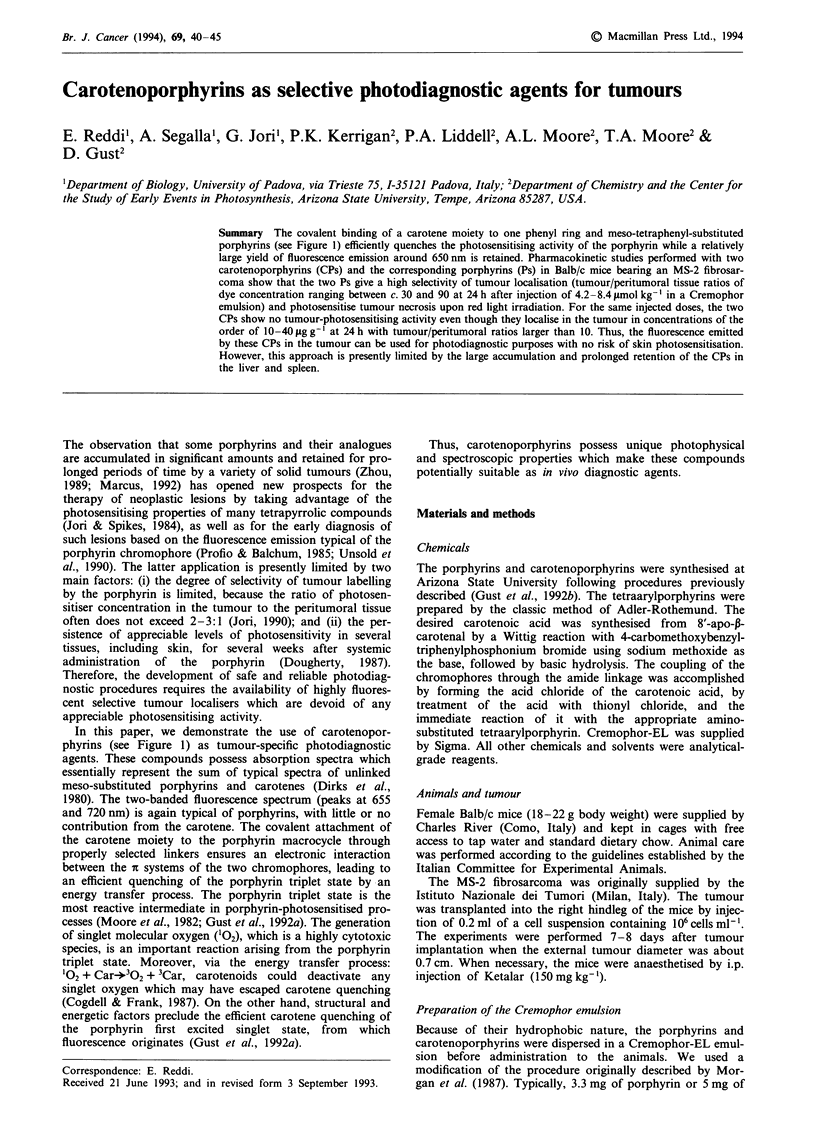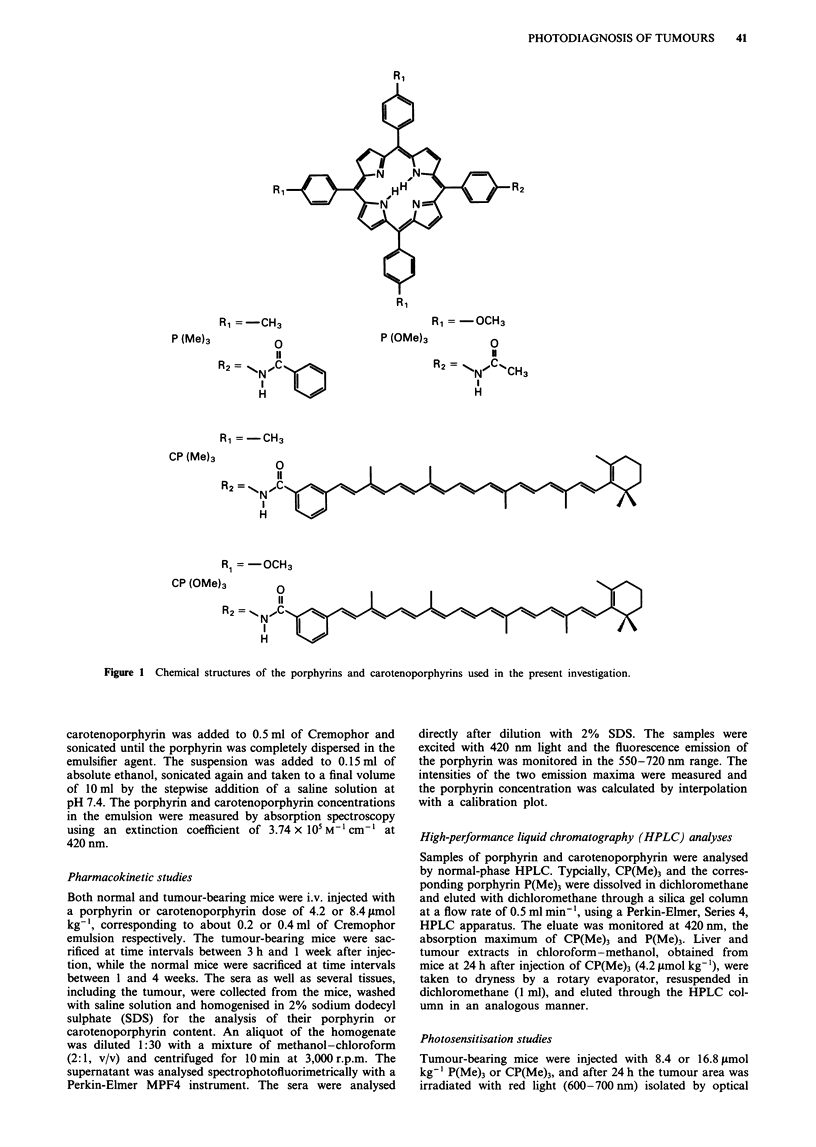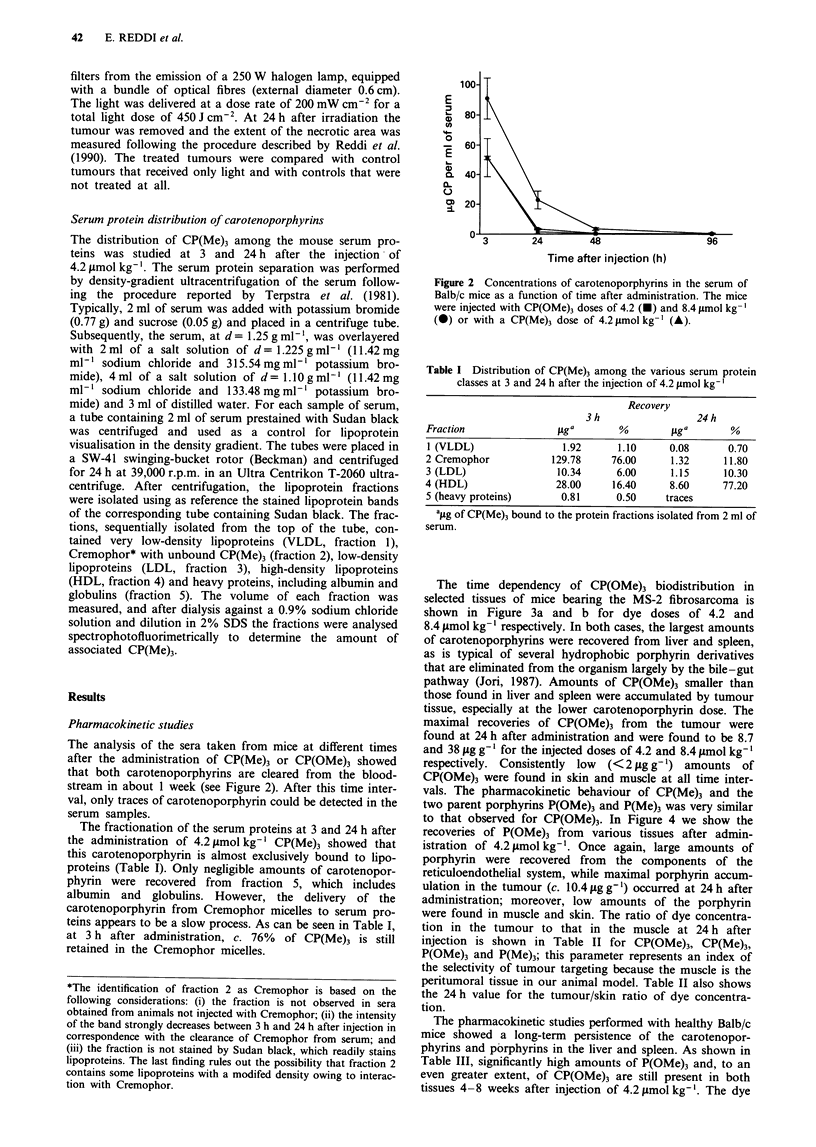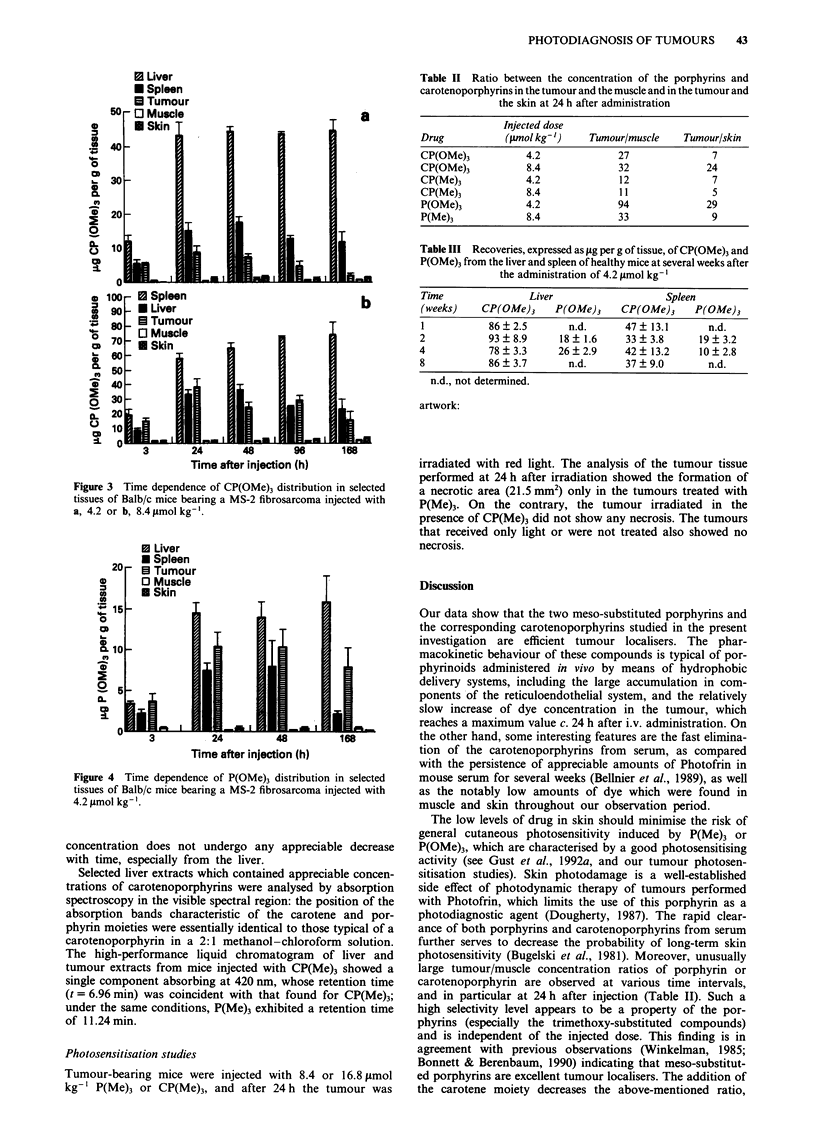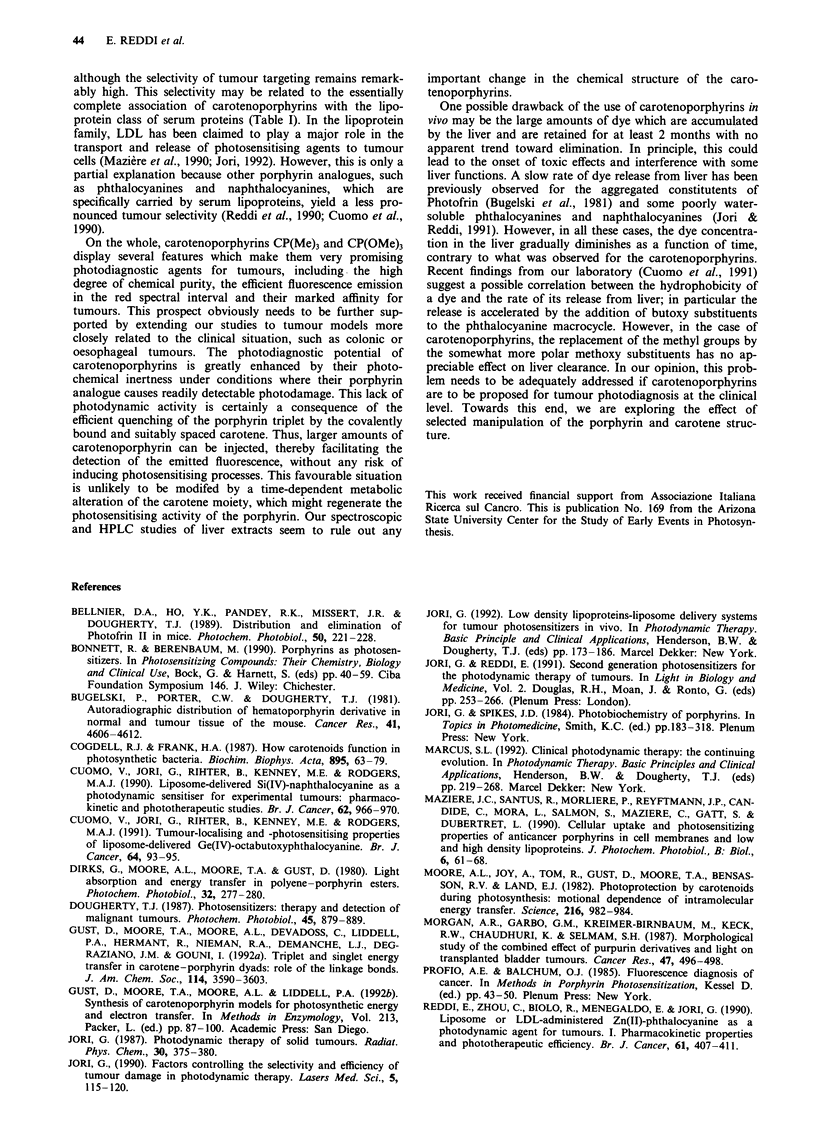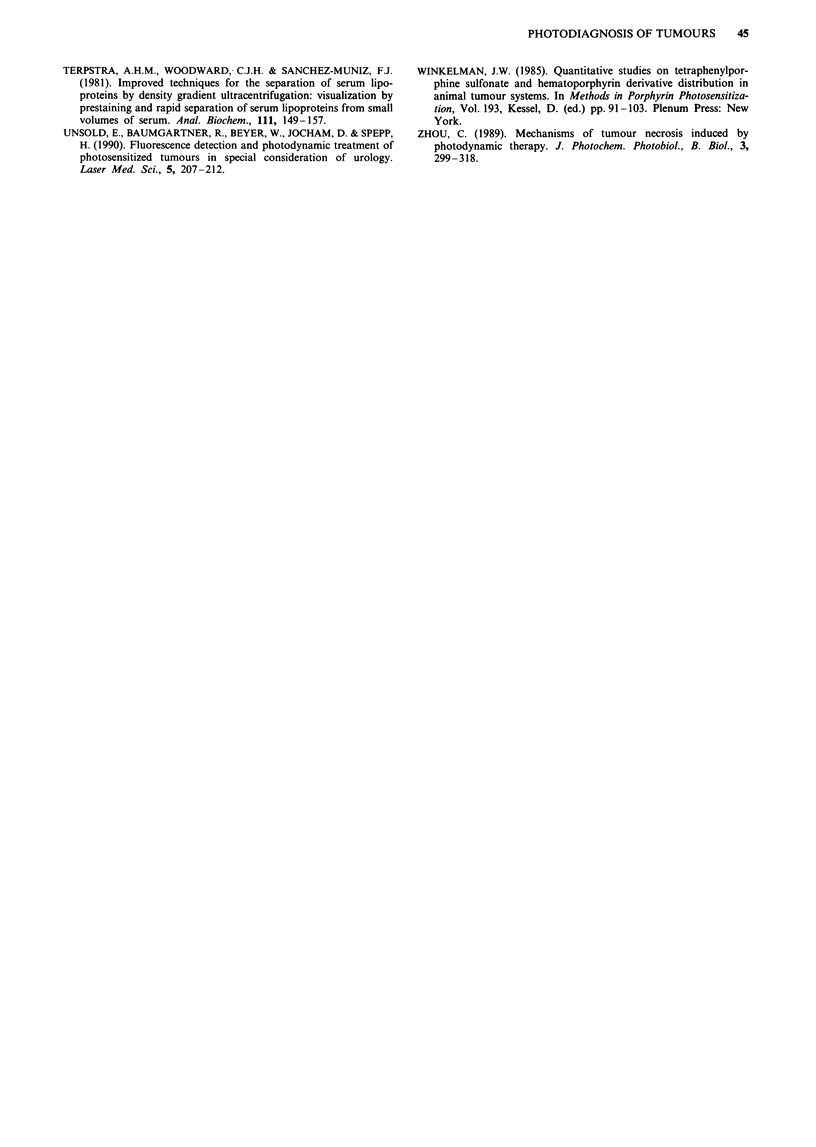# Carotenoporphyrins as selective photodiagnostic agents for tumours.

**DOI:** 10.1038/bjc.1994.6

**Published:** 1994-01

**Authors:** E. Reddi, A. Segalla, G. Jori, P. K. Kerrigan, P. A. Liddell, A. L. Moore, T. A. Moore, D. Gust

**Affiliations:** Department of Biology, University of Padova, Italy.

## Abstract

The covalent binding of a carotene moiety to one phenyl ring and meso-tetraphenyl-substituted porphyrins (see Figure 1) efficiently quenches the photosensitising activity of the porphyrin while a relatively large yield of fluorescence emission around 650 nm is retained. Pharmacokinetic studies performed with two carotenoporphyrins (CPs) and the corresponding porphyrins (Ps) in Balb/c mice bearing an MS-2 fibrosarcoma show that the two Ps give a high selectivity of tumour localisation (tumour/peritumoral tissue ratios of dye concentration ranging between c. 30 and 90 at 24 h after injection of 4.2-8.4 mumol kg-1 in a Cremophor emulsion) and photosensitive tumour necrosis upon red light irradiation. For the same injected doses, the two CPs show no tumour-photosensitising activity even though they localise in the tumour in concentrations of the order of 10-40 micrograms g-1 at 24 h with tumour/peritumoral ratios larger than 10. Thus, the fluorescence emitted by these CPs in the tumour can be used for photodiagnostic purposes with no risk of skin photosensitisation. However, this approach is presently limited by the large accumulation and prolonged retention of the CPs in the liver and spleen.


Br. J. Cancer (1994), 69, 40–45 © Macmillan Press Ltd., 1994

### Carotenoporphyrins as selective photodiagnostic agents for tumours

E. Reddi[1], A. Segalla[1], G. Jori[1], P.K. Kerrigan[2], P.A. Liddell[2], A.L. Moore[2], T.A. Moore[2] & D. Gust[2]

[1]*Department of Biology, University of Padova, via Trieste 75, I-35121 Padova, Italy;* [2]*Department of Chemistry and the Center for the Study of Early Events in Photosynthesis, Arizona State University, Tempe, Arizona 85287, USA.*

**Summary** The covalent binding of a carotene moiety to one phenyl ring and meso-tetraphenyl-substituted porphyrins (see Figure 1) efficiently quenches the photosensitising activity of the porphyrin while a relatively large yield of fluorescence emission around 650 nm is retained. Pharmacokinetic studies performed with two carotenoporphyrins (CPs) and the corresponding porphyrins (Ps) in Balb/c mice bearing an MS-2 fibrosarcoma show that the two Ps give a high selectivity of tumour localisation (tumour/peritumoral tissue ratios of dye concentration ranging between *c.* 30 and 90 at 24 h after injection of 4.2–8.4 $\mu$mol kg$^{-1}$ in a Cremophor emulsion) and photosensitise tumour necrosis upon red light irradiation. For the same injected doses, the two CPs show no tumour-photosensitising activity even though they localise in the tumour in concentrations of the order of 10–40 $\mu$g g$^{-1}$ at 24 h with tumour/peritumoral ratios larger than 10. Thus, the fluorescence emitted by these CPs in the tumour can be used for photodiagnostic purposes with no risk of skin photosensitisation. However, this approach is presently limited by the large accumulation and prolonged retention of the CPs in the liver and spleen.

The observation that some porphyrins and their analogues are accumulated in significant amounts and retained for prolonged periods of time by a variety of solid tumours (Zhou, 1989; Marcus, 1992) has opened new prospects for the therapy of neoplastic lesions by taking advantage of the photosensitising properties of many tetrapyrrolic compounds (Jori & Spikes, 1984), as well as for the early diagnosis of such lesions based on the fluorescence emission typical of the porphyrin chromophore (Profio & Balchum, 1985; Unsold *et al.*, 1990). The latter application is presently limited by two main factors: (i) the degree of selectivity of tumour labelling by the porphyrin is limited, because the ratio of photosensitiser concentration in the tumour to the peritumoral tissue often does not exceed 2–3:1 (Jori, 1990); and (ii) the persistence of appreciable levels of photosensitivity in several tissues, including skin, for several weeks after systemic administration of the porphyrin (Dougherty, 1987). Therefore, the development of safe and reliable photodiagnostic procedures requires the availability of highly fluorescent selective tumour localisers which are devoid of any appreciable photosensitising activity.

In this paper, we demonstrate the use of carotenoporphyrins (see Figure 1) as tumour-specific photodiagnostic agents. These compounds possess absorption spectra which essentially represent the sum of typical spectra of unlinked meso-substituted porphyrins and carotenes (Dirks *et al.*, 1980). The two-banded fluorescence spectrum (peaks at 655 and 720 nm) is again typical of porphyrins, with little or no contribution from the carotene. The covalent attachment of the carotene moiety to the porphyrin macrocycle through properly selected linkers ensures an electronic interaction between the $\pi$ systems of the two chromophores, leading to an efficient quenching of the porphyrin triplet state by an energy transfer process. The porphyrin triplet state is the most reactive intermediate in porphyrin-photosensitised processes (Moore *et al.*, 1982; Gust *et al.*, 1992*a*). The generation of singlet molecular oxygen ($^1O_2$), which is a highly cytotoxic species, is an important reaction arising from the porphyrin triplet state. Moreover, via the energy transfer process: $^1O_2 + Car \rightarrow {}^3O_2 + {}^3Car$, carotenoids could deactivate any singlet oxygen which may have escaped carotene quenching (Cogdell & Frank, 1987). On the other hand, structural and energetic factors preclude the efficient carotene quenching of the porphyrin first excited singlet state, from which fluorescence originates (Gust *et al.*, 1992*a*).

Correspondence: E. Reddi.
Received 21 June 1993; and in revised form 3 September 1993.

Thus, carotenoporphyrins possess unique photophysical and spectroscopic properties which make these compounds potentially suitable as *in vivo* diagnostic agents.

### Materials and methods

### *Chemicals*

The porphyrins and carotenoporphyrins were synthesised at Arizona State University following procedures previously described (Gust *et al.*, 1992*b*). The tetraarylporphyrins were prepared by the classic method of Adler-Rothemund. The desired carotenoic acid was synthesised from 8′-apo-$\beta$-carotenal by a Wittig reaction with 4-carbomethoxybenzyltriphenylphosphonium bromide using sodium methoxide as the base, followed by basic hydrolysis. The coupling of the chromophores through the amide linkage was accomplished by forming the acid chloride of the carotenoic acid, by treatment of the acid with thionyl chloride, and the immediate reaction of it with the appropriate amino-substituted tetraarylporphyrin. Cremophor-EL was supplied by Sigma. All other chemicals and solvents were analytical-grade reagents.

### *Animals and tumour*

Female Balb/c mice (18–22 g body weight) were supplied by Charles River (Como, Italy) and kept in cages with free access to tap water and standard dietary chow. Animal care was performed according to the guidelines established by the Italian Committee for Experimental Animals.

The MS-2 fibrosarcoma was originally supplied by the Istituto Nazionale dei Tumori (Milan, Italy). The tumour was transplanted into the right hindleg of the mice by injection of 0.2 ml of a cell suspension containing $10^6$ cells ml$^{-1}$. The experiments were performed 7–8 days after tumour implantation when the external tumour diameter was about 0.7 cm. When necessary, the mice were anaesthetised by i.p. injection of Ketalar (150 mg kg$^{-1}$).

### *Preparation of the Cremophor emulsion*

Because of their hydrophobic nature, the porphyrins and carotenoporphyrins were dispersed in a Cremophor-EL emulsion before administration to the animals. We used a modification of the procedure originally described by Morgan *et al.* (1987). Typically, 3.3 mg of porphyrin or 5 mg of

**Figure 1** Chemical structures of the porphyrins and carotenoporphyrins used in the present investigation.

carotenoporphyrin was added to 0.5 ml of Cremophor and sonicated until the porphyrin was completely dispersed in the emulsifier agent. The suspension was added to 0.15 ml of absolute ethanol, sonicated again and taken to a final volume of 10 ml by the stepwise addition of a saline solution at pH 7.4. The porphyrin and carotenoporphyrin concentrations in the emulsion were measured by absorption spectroscopy using an extinction coefficient of $3.74 \times 10^5\ \mathrm{M}^{-1}\ \mathrm{cm}^{-1}$ at 420 nm.

*Pharmacokinetic studies*

Both normal and tumour-bearing mice were i.v. injected with a porphyrin or carotenoporphyrin dose of 4.2 or 8.4 μmol $\mathrm{kg}^{-1}$, corresponding to about 0.2 or 0.4 ml of Cremophor emulsion respectively. The tumour-bearing mice were sacrificed at time intervals between 3 h and 1 week after injection, while the normal mice were sacrificed at time intervals between 1 and 4 weeks. The sera as well as several tissues, including the tumour, were collected from the mice, washed with saline solution and homogenised in 2% sodium dodecyl sulphate (SDS) for the analysis of their porphyrin or carotenoporphyrin content. An aliquot of the homogenate was diluted 1:30 with a mixture of methanol–chloroform (2:1, v/v) and centrifuged for 10 min at 3,000 r.p.m. The supernatant was analysed spectrophotofluorimetrically with a Perkin-Elmer MPF4 instrument. The sera were analysed directly after dilution with 2% SDS. The samples were excited with 420 nm light and the fluorescence emission of the porphyrin was monitored in the 550–720 nm range. The intensities of the two emission maxima were measured and the porphyrin concentration was calculated by interpolation with a calibration plot.

*High-performance liquid chromatography (HPLC) analyses*

Samples of porphyrin and carotenoporphyrin were analysed by normal-phase HPLC. Typcially, $\mathrm{CP(Me)_3}$ and the corresponding porphyrin $\mathrm{P(Me)_3}$ were dissolved in dichloromethane and eluted with dichloromethane through a silica gel column at a flow rate of 0.5 ml $\mathrm{min}^{-1}$, using a Perkin-Elmer, Series 4, HPLC apparatus. The eluate was monitored at 420 nm, the absorption maximum of $\mathrm{CP(Me)_3}$ and $\mathrm{P(Me)_3}$. Liver and tumour extracts in chloroform–methanol, obtained from mice at 24 h after injection of $\mathrm{CP(Me)_3}$ (4.2 μmol $\mathrm{kg}^{-1}$), were taken to dryness by a rotary evaporator, resuspended in dichloromethane (1 ml), and eluted through the HPLC column in an analogous manner.

*Photosensitisation studies*

Tumour-bearing mice were injected with 8.4 or 16.8 μmol $\mathrm{kg}^{-1}$ $\mathrm{P(Me)_3}$ or $\mathrm{CP(Me)_3}$, and after 24 h the tumour area was irradiated with red light (600–700 nm) isolated by optical filters from the emission of a 250 W halogen lamp, equipped with a bundle of optical fibres (external diameter 0.6 cm). The light was delivered at a dose rate of 200 mW $cm^{-2}$ for a total light dose of 450 J $cm^{-2}$. At 24 h after irradiation the tumour was removed and the extent of the necrotic area was measured following the procedure described by Reddi et al. (1990). The treated tumours were compared with control tumours that received only light and with controls that were not treated at all.

*Serum protein distribution of carotenoporphyrins*

The distribution of $CP(Me)_3$ among the mouse serum proteins was studied at 3 and 24 h after the injection of 4.2 μmol $kg^{-1}$. The serum protein separation was performed by density-gradient ultracentrifugation of the serum following the procedure reported by Terpstra et al. (1981). Typically, 2 ml of serum was added with potassium bromide (0.77 g) and sucrose (0.05 g) and placed in a centrifuge tube. Subsequently, the serum, at $d = 1.25$ g $ml^{-1}$, was overlayered with 2 ml of a salt solution of $d = 1.225$ g $ml^{-1}$ (11.42 mg $ml^{-1}$ sodium chloride and 315.54 mg $ml^{-1}$ potassium bromide), 4 ml of a salt solution of $d = 1.10$ g $ml^{-1}$ (11.42 mg $ml^{-1}$ sodium chloride and 133.48 mg $ml^{-1}$ potassium bromide) and 3 ml of distilled water. For each sample of serum, a tube containing 2 ml of serum prestained with Sudan black was centrifuged and used as a control for lipoprotein visualisation in the density gradient. The tubes were placed in a SW-41 swinging-bucket rotor (Beckman) and centrifuged for 24 h at 39,000 r.p.m. in an Ultra Centrikon T-2060 ultracentrifuge. After centrifugation, the lipoprotein fractions were isolated using as reference the stained lipoprotein bands of the corresponding tube containing Sudan black. The fractions, sequentially isolated from the top of the tube, contained very low-density lipoproteins (VLDL, fraction 1), Cremophor* with unbound $CP(Me)_3$ (fraction 2), low-density lipoproteins (LDL, fraction 3), high-density lipoproteins (HDL, fraction 4) and heavy proteins, including albumin and globulins (fraction 5). The volume of each fraction was measured, and after dialysis against a 0.9% sodium chloride solution and dilution in 2% SDS the fractions were analysed spectrophotofluorimetrically to determine the amount of associated $CP(Me)_3$.

### Results

*Pharmacokinetic studies*

The analysis of the sera taken from mice at different times after the administration of $CP(Me)_3$ or $CP(OMe)_3$ showed that both carotenoporphyrins are cleared from the bloodstream in about 1 week (see Figure 2). After this time interval, only traces of carotenoporphyrin could be detected in the serum samples.

The fractionation of the serum proteins at 3 and 24 h after the administration of 4.2 μmol $kg^{-1}$ $CP(Me)_3$ showed that this carotenoporphyrin is almost exclusively bound to lipoproteins (Table I). Only negligible amounts of carotenoporphyrin were recovered from fraction 5, which includes albumin and globulins. However, the delivery of the carotenoporphyrin from Cremophor micelles to serum proteins appears to be a slow process. As can be seen in Table I, at 3 h after administration, c. 76% of $CP(Me)_3$ is still retained in the Cremophor micelles.

*The identification of fraction 2 as Cremophor is based on the following considerations: (i) the fraction is not observed in sera obtained from animals not injected with Cremophor; (ii) the intensity of the band strongly decreases between 3 h and 24 h after injection in correspondence with the clearance of Cremophor from serum; and (iii) the fraction is not stained by Sudan black, which readily stains lipoproteins. The last finding rules out the possibility that fraction 2 contains some lipoproteins with a modifed density owing to interaction with Cremophor.

**Figure 2** Concentrations of carotenoporphyrins in the serum of Balb/c mice as a function of time after administration. The mice were injected with $CP(OMe)_3$ doses of 4.2 (■) and 8.4 μmol $kg^{-1}$ (●) or with a $CP(Me)_3$ dose of 4.2 μmol $kg^{-1}$ (▲).

**Table I** Distribution of $CP(Me)_3$ among the various serum protein classes at 3 and 24 h after the injection of 4.2 μmol $kg^{-1}$

| | Recovery | | | |
|---|---|---|---|---|
| | 3 h | | 24 h | |
| Fraction | μg[a] | % | μg[a] | % |
| 1 (VLDL) | 1.92 | 1.10 | 0.08 | 0.70 |
| 2 Cremophor | 129.78 | 76.00 | 1.32 | 11.80 |
| 3 (LDL) | 10.34 | 6.00 | 1.15 | 10.30 |
| 4 (HDL) | 28.00 | 16.40 | 8.60 | 77.20 |
| 5 (heavy proteins) | 0.81 | 0.50 | traces | |

[a]μg of $CP(Me)_3$ bound to the protein fractions isolated from 2 ml of serum.

The time dependency of $CP(OMe)_3$ biodistribution in selected tissues of mice bearing the MS-2 fibrosarcoma is shown in Figure 3a and b for dye doses of 4.2 and 8.4 μmol $kg^{-1}$ respectively. In both cases, the largest amounts of carotenoporphyrins were recovered from liver and spleen, as is typical of several hydrophobic porphyrin derivatives that are eliminated from the organism largely by the bile–gut pathway (Jori, 1987). Amounts of $CP(OMe)_3$ smaller than those found in liver and spleen were accumulated by tumour tissue, especially at the lower carotenoporphyrin dose. The maximal recoveries of $CP(OMe)_3$ from the tumour were found at 24 h after administration and were found to be 8.7 and 38 μg $g^{-1}$ for the injected doses of 4.2 and 8.4 μmol $kg^{-1}$ respectively. Consistently low (<2 μg $g^{-1}$) amounts of $CP(OMe)_3$ were found in skin and muscle at all time intervals. The pharmacokinetic behaviour of $CP(Me)_3$ and the two parent porphyrins $P(OMe)_3$ and $P(Me)_3$ was very similar to that observed for $CP(OMe)_3$. In Figure 4 we show the recoveries of $P(OMe)_3$ from various tissues after administration of 4.2 μmol $kg^{-1}$. Once again, large amounts of porphyrin were recovered from the components of the reticuloendothelial system, while maximal porphyrin accumulation in the tumour (c. 10.4 μg $g^{-1}$) occurred at 24 h after administration; moreover, low amounts of the porphyrin were found in muscle and skin. The ratio of dye concentration in the tumour to that in the muscle at 24 h after injection is shown in Table II for $CP(OMe)_3$, $CP(Me)_3$, $P(OMe)_3$ and $P(Me)_3$; this parameter represents an index of the selectivity of tumour targeting because the muscle is the peritumoral tissue in our animal model. Table II also shows the 24 h value for the tumour/skin ratio of dye concentration.

The pharmacokinetic studies performed with healthy Balb/c mice showed a long-term persistence of the carotenoporphyrins and porphyrins in the liver and spleen. As shown in Table III, significantly high amounts of $P(OMe)_3$ and, to an even greater extent, of $CP(OMe)_3$ are still present in both tissues 4–8 weeks after injection of 4.2 μmol $kg^{-1}$. The dye

**Figure 3** Time dependence of $CP(OMe)_3$ distribution in selected tissues of Balb/c mice bearing a MS-2 fibrosarcoma injected with **a**, 4.2 or **b**, 8.4 μmol $kg^{-1}$.

**Figure 4** Time dependence of $P(OMe)_3$ distribution in selected tissues of Balb/c mice bearing a MS-2 fibrosarcoma injected with 4.2 μmol $kg^{-1}$.

concentration does not undergo any appreciable decrease with time, especially from the liver.

Selected liver extracts which contained appreciable concentrations of carotenoporphyrins were analysed by absorption spectroscopy in the visible spectral region: the position of the absorption bands characteristic of the carotene and porphyrin moieties were essentially identical to those typical of a carotenoporphyrin in a 2:1 methanol–chloroform solution. The high-performance liquid chromatogram of liver and tumour extracts from mice injected with $CP(Me)_3$ showed a single component absorbing at 420 nm, whose retention time ($t = 6.96$ min) was coincident with that found for $CP(Me)_3$; under the same conditions, $P(Me)_3$ exhibited a retention time of 11.24 min.

*Photosensitisation studies*

Tumour-bearing mice were injected with 8.4 or 16.8 μmol $kg^{-1}$ $P(Me)_3$ or $CP(Me)_3$, and after 24 h the tumour was

**Table II** Ratio between the concentration of the porphyrins and carotenoporphyrins in the tumour and the muscle and in the tumour and the skin at 24 h after administration

| *Drug* | *Injected dose (μmol kg⁻¹)* | *Tumour/muscle* | *Tumour/skin* |
|---|---|---|---|
| $CP(OMe)_3$ | 4.2 | 27 | 7 |
| $CP(OMe)_3$ | 8.4 | 32 | 24 |
| $CP(Me)_3$ | 4.2 | 12 | 7 |
| $CP(Me)_3$ | 8.4 | 11 | 5 |
| $P(OMe)_3$ | 4.2 | 94 | 29 |
| $P(Me)_3$ | 8.4 | 33 | 9 |

**Table III** Recoveries, expressed as μg per g of tissue, of $CP(OMe)_3$ and $P(OMe)_3$ from the liver and spleen of healthy mice at several weeks after the administration of 4.2 μmol $kg^{-1}$

| *Time* | *Liver* | | *Spleen* | |
|---|---|---|---|---|
| *(weeks)* | *CP(OMe)₃* | *P(OMe)₃* | *CP(OMe)₃* | *P(OMe)₃* |
| 1 | 86 ± 2.5 | n.d. | 47 ± 13.1 | n.d. |
| 2 | 93 ± 8.9 | 18 ± 1.6 | 33 ± 3.8 | 19 ± 3.2 |
| 4 | 78 ± 3.3 | 26 ± 2.9 | 42 ± 13.2 | 10 ± 2.8 |
| 8 | 86 ± 3.7 | n.d. | 37 ± 9.0 | n.d. |

n.d., not determined.

artwork:

irradiated with red light. The analysis of the tumour tissue performed at 24 h after irradiation showed the formation of a necrotic area (21.5 $mm^2$) only in the tumours treated with $P(Me)_3$. On the contrary, the tumour irradiated in the presence of $CP(Me)_3$ did not show any necrosis. The tumours that received only light or were not treated also showed no necrosis.

### Discussion

Our data show that the two meso-substituted porphyrins and the corresponding carotenoporphyrins studied in the present investigation are efficient tumour localisers. The pharmacokinetic behaviour of these compounds is typical of porphyrinoids administered *in vivo* by means of hydrophobic delivery systems, including the large accumulation in components of the reticuloendothelial system, and the relatively slow increase of dye concentration in the tumour, which reaches a maximum value *c*. 24 h after i.v. administration. On the other hand, some interesting features are the fast elimination of the carotenoporphyrins from serum, as compared with the persistence of appreciable amounts of Photofrin in mouse serum for several weeks (Bellnier *et al*., 1989), as well as the notably low amounts of dye which were found in muscle and skin throughout our observation period.

The low levels of drug in skin should minimise the risk of general cutaneous photosensitivity induced by $P(Me)_3$ or $P(OMe)_3$, which are characterised by a good photosensitising activity (see Gust *et al*., 1992*a*, and our tumour photosensitisation studies). Skin photodamage is a well-established side effect of photodynamic therapy of tumours performed with Photofrin, which limits the use of this porphyrin as a photodiagnostic agent (Dougherty, 1987). The rapid clearance of both porphyrins and carotenoporphyrins from serum further serves to decrease the probability of long-term skin photosensitivity (Bugelski *et al*., 1981). Moreover, unusually large tumour/muscle concentration ratios of porphyrin or carotenoporphyrin are observed at various time intervals, and in particular at 24 h after injection (Table II). Such a high selectivity level appears to be a property of the porphyrins (especially the trimethoxy-substituted compounds) and is independent of the injected dose. This finding is in agreement with previous observations (Winkelman, 1985; Bonnett & Berenbaum, 1990) indicating that meso-substituted porphyrins are excellent tumour localisers. The addition of the carotene moiety decreases the above-mentioned ratio, although the selectivity of tumour targeting remains remarkably high. This selectivity may be related to the essentially complete association of carotenoporphyrins with the lipoprotein class of serum proteins (Table I). In the lipoprotein family, LDL has been claimed to play a major role in the transport and release of photosensitising agents to tumour cells (Mazière et al., 1990; Jori, 1992). However, this is only a partial explanation because other porphyrin analogues, such as phthalocyanines and naphthalocyanines, which are specifically carried by serum lipoproteins, yield a less pronounced tumour selectivity (Reddi et al., 1990; Cuomo et al., 1990).

On the whole, carotenoporphyrins $CP(Me)_3$ and $CP(OMe)_3$ display several features which make them very promising photodiagnostic agents for tumours, including the high degree of chemical purity, the efficient fluorescence emission in the red spectral interval and their marked affinity for tumours. This prospect obviously needs to be further supported by extending our studies to tumour models more closely related to the clinical situation, such as colonic or oesophageal tumours. The photodiagnostic potential of carotenoporphyrins is greatly enhanced by their photochemical inertness under conditions where their porphyrin analogue causes readily detectable photodamage. This lack of photodynamic activity is certainly a consequence of the efficient quenching of the porphyrin triplet by the covalently bound and suitably spaced carotene. Thus, larger amounts of carotenoporphyrin can be injected, thereby facilitating the detection of the emitted fluorescence, without any risk of inducing photosensitising processes. This favourable situation is unlikely to be modifed by a time-dependent metabolic alteration of the carotene moiety, which might regenerate the photosensitising activity of the porphyrin. Our spectroscopic and HPLC studies of liver extracts seem to rule out any important change in the chemical structure of the carotenoporphyrins.

One possible drawback of the use of carotenoporphyrins *in vivo* may be the large amounts of dye which are accumulated by the liver and are retained for at least 2 months with no apparent trend toward elimination. In principle, this could lead to the onset of toxic effects and interference with some liver functions. A slow rate of dye release from liver has been previously observed for the aggregated constitutents of Photofrin (Bugelski et al., 1981) and some poorly water-soluble phthalocyanines and naphthalocyanines (Jori & Reddi, 1991). However, in all these cases, the dye concentration in the liver gradually diminishes as a function of time, contrary to what was observed for the carotenoporphyrins. Recent findings from our laboratory (Cuomo et al., 1991) suggest a possible correlation between the hydrophobicity of a dye and the rate of its release from liver; in particular the release is accelerated by the addition of butoxy substituents to the phthalocyanine macrocycle. However, in the case of carotenoporphyrins, the replacement of the methyl groups by the somewhat more polar methoxy substituents has no appreciable effect on liver clearance. In our opinion, this problem needs to be adequately addressed if carotenoporphyrins are to be proposed for tumour photodiagnosis at the clinical level. Towards this end, we are exploring the effect of selected manipulation of the porphyrin and carotene structure.

This work received financial support from Associazione Italiana Ricerca sul Cancro. This is publication No. 169 from the Arizona State University Center for the Study of Early Events in Photosynthesis.

### References

BELLNIER, D.A., HO, Y.K., PANDEY, R.K., MISSERT, J.R. & DOUGHERTY, T.J. (1989). Distribution and elimination of Photofrin II in mice. *Photochem. Photobiol.*, **50**, 221-228.

BONNETT, R. & BERENBAUM, M. (1990). Porphyrins as photosensitizers. In *Photosensitizing Compounds: Their Chemistry, Biology and Clinical Use*, Bock, G. & Harnett, S. (eds) pp. 40-59. Ciba Foundation Symposium 146. J. Wiley: Chichester.

BUGELSKI, P., PORTER, C.W. & DOUGHERTY, T.J. (1981). Autoradiographic distribution of hematoporphyrin derivative in normal and tumour tissue of the mouse. *Cancer Res.*, **41**, 4606-4612.

COGDELL, R.J. & FRANK, H.A. (1987). How carotenoids function in photosynthetic bacteria. *Biochim. Biophys. Acta*, **895**, 63-79.

CUOMO, V., JORI, G., RIHTER, B., KENNEY, M.E. & RODGERS, M.A.J. (1990). Liposome-delivered Si(IV)-naphthalocyanine as a photodynamic sensitiser for experimental tumours: pharmacokinetic and phototherapeutic studies. *Br. J. Cancer*, **62**, 966-970.

CUOMO, V., JORI, G., RIHTER, B., KENNEY, M.E. & RODGERS, M.A.J. (1991). Tumour-localising and -photosensitising properties of liposome-delivered Ge(IV)-octabutoxyphthalocyanine. *Br. J. Cancer*, **64**, 93-95.

DIRKS, G., MOORE, A.L., MOORE, T.A. & GUST, D. (1980). Light absorption and energy transfer in polyene-porphyrin esters. *Photochem. Photobiol.*, **32**, 277-280.

DOUGHERTY, T.J. (1987). Photosensitizers: therapy and detection of malignant tumours. *Photochem. Photobiol.*, **45**, 879-889.

GUST, D., MOORE, T.A., MOORE, A.L., DEVADOSS, C., LIDDELL, P.A., HERMANT, R., NIEMAN, R.A., DEMANCHE, L.J., DEGRAZIANO, J.M. & GOUNI, I. (1992*a*). Triplet and singlet energy transfer in carotene-porphyrin dyads: role of the linkage bonds. *J. Am. Chem. Soc.*, **114**, 3590-3603.

GUST, D., MOORE, T.A., MOORE, A.L. & LIDDELL, P.A. (1992*b*). Synthesis of carotenoporphyrin models for photosynthetic energy and electron transfer. In *Methods in Enzymology*, Vol. 213, Packer, L. (ed.) pp. 87-100. Academic Press: San Diego.

JORI, G. (1987). Photodynamic therapy of solid tumours. *Radiat. Phys. Chem.*, **30**, 375-380.

JORI, G., (1990). Factors controlling the selectivity and efficiency of tumour damage in photodynamic therapy. *Lasers Med. Sci.*, **5**, 115-120.

JORI, G. (1992). Low density lipoproteins-liposome delivery systems for tumour photosensitizers in vivo. In *Photodynamic Therapy. Basic Principle and Clinical Applications*, Henderson, B.W. & Dougherty, T.J. (eds) pp. 173-186. Marcel Dekker: New York.

JORI, G. & REDDI, E. (1991). Second generation photosensitizers for the photodynamic therapy of tumours. In *Light in Biology and Medicine*, Vol. 2. Douglas, R.H., Moan, J. & Ronto, G. (eds) pp. 253-266. (Plenum Press: London).

JORI, G. & SPIKES, J.D. (1984). Photobiochemistry of porphyrins. In *Topics in Photomedicine*, Smith, K.C. (ed.) pp.183-318. Plenum Press: New York.

MARCUS, S.L. (1992). Clinical photodynamic therapy: the continuing evolution. In *Photodynamic Therapy. Basic Principles and Clinical Applications*, Henderson, B.W. & Dougherty, T.J. (eds) pp. 219-268. Marcel Dekker: New York.

MAZIERE, J.C., SANTUS, R., MORLIERE, P., REYFTMANN, J.P., CANDIDE, C., MORA, L., SALMON, S., MAZIERE, C., GATT, S. & DUBERTRET, L. (1990). Cellular uptake and photosensitizing properties of anticancer porphyrins in cell membranes and low and high density lipoproteins. *J. Photochem. Photobiol., B: Biol.*, **6**, 61-68.

MOORE, A.L., JOY, A., TOM, R., GUST, D., MOORE, T.A., BENSASSON, R.V. & LAND, E.J. (1982). Photoprotection by carotenoids during photosynthesis: motional dependence of intramolecular energy transfer. *Science*, **216**, 982-984.

MORGAN, A.R., GARBO, G.M., KREIMER-BIRNBAUM, M., KECK, R.W., CHAUDHURI, K. & SELMAM, S.H. (1987). Morphological study of the combined effect of purpurin derivatives and light on transplanted bladder tumours. *Cancer Res.*, **47**, 496-498.

PROFIO, A.E. & BALCHUM, O.J. (1985). Fluorescence diagnosis of cancer. In *Methods in Porphyrin Photosensitization*, Kessel D. (ed.) pp. 43-50. Plenum Press: New York.

REDDI, E., ZHOU, C., BIOLO, R., MENEGALDO, E. & JORI, G. (1990). Liposome or LDL-administered Zn(II)-phthalocyanine as a photodynamic agent for tumours. I. Pharmacokinetic properties and phototherapeutic efficiency. *Br. J. Cancer*, **61**, 407-411.

TERPSTRA, A.H.M., WOODWARD, C.J.H. & SANCHEZ-MUNIZ, F.J. (1981). Improved techniques for the separation of serum lipoproteins by density gradient ultracentrifugation: visualization by prestaining and rapid separation of serum lipoproteins from small volumes of serum. *Anal. Biochem.*, **111**, 149–157.

UNSOLD, E., BAUMGARTNER, R., BEYER, W., JOCHAM, D. & SPEPP, H. (1990). Fluorescence detection and photodynamic treatment of photosensitized tumours in special consideration of urology. *Laser Med. Sci.*, **5**, 207–212.

WINKELMAN, J.W. (1985). Quantitative studies on tetraphenylporphine sulfonate and hematoporphyrin derivative distribution in animal tumour systems. In *Methods in Porphyrin Photosensitization*, Vol. 193, Kessel, D. (ed.) pp. 91–103. Plenum Press: New York.

ZHOU, C. (1989). Mechanisms of tumour necrosis induced by photodynamic therapy. *J. Photochem. Photobiol., B. Biol.*, **3**, 299–318.