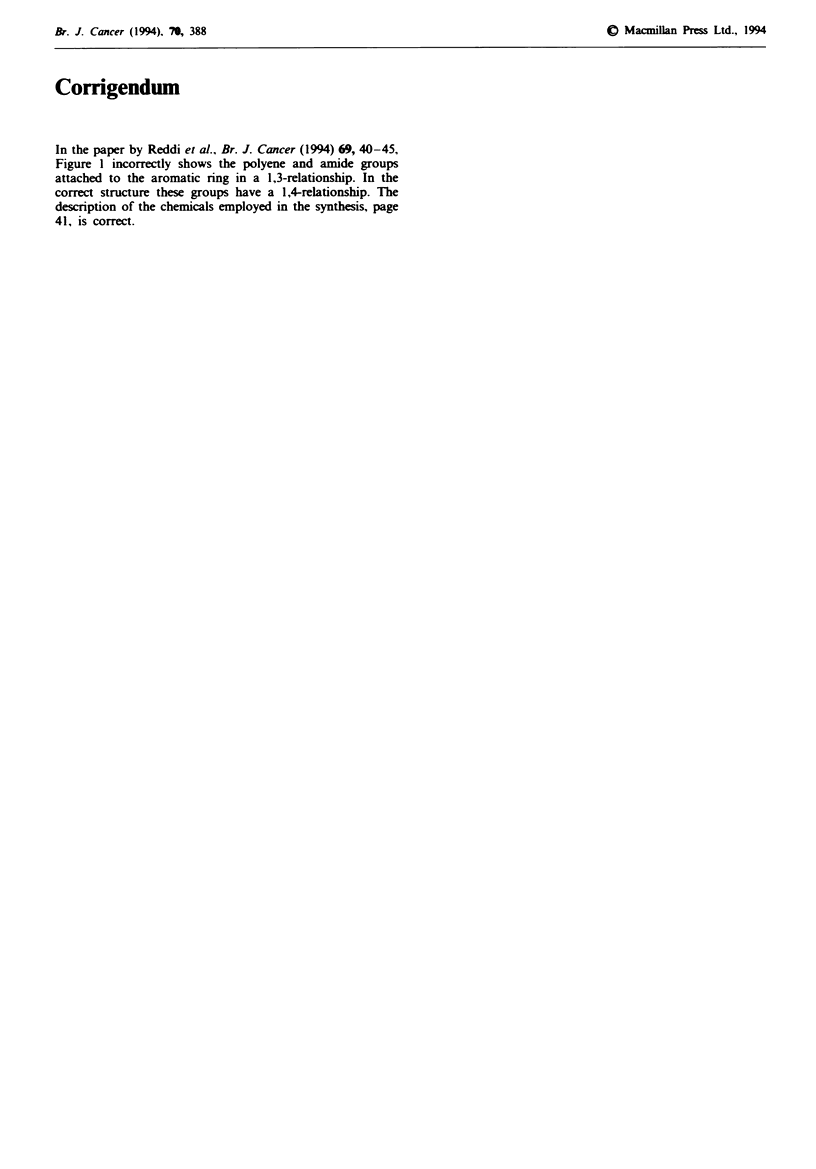# Corrigendum

**Published:** 1994-08

**Authors:** 


 © Macmillan Press Ltd., 1994

### Corrigendum

In the paper by Reddi *et al.*, *Br. J. Cancer* (1994) **69**, 40–45, Figure 1 incorrectly shows the polyene and amide groups attached to the aromatic ring in a 1,3-relationship. In the correct structure these groups have a 1,4-relationship. The description of the chemicals employed in the synthesis, page 41, is correct.